# Risk Factors for Oropharyngeal Dysphagia in Older Adults - Evidence from Consorci Sanitari Del Maresme and Mataró University Hospital, Catalonia, Spain

**DOI:** 10.14336/AD.2025.0345

**Published:** 2026-05-21

**Authors:** Lucilla Guidotti, Jorge Iván Castañeda-Maldonado, Marta Santiago, Pere Clavé, Omar Ortega

**Affiliations:** ^1^Gastrointestinal Physiology Laboratory, Department of Surgery, Hospital Universitari de Mataró, Consorci Sanitari del Maresme Universitat Autònoma de Barcelona, Barcelona, Spain.; ^2^Germans Trias i Pujol Research Institute (IGTP).; ^3^Centro de Investigación Biomédica en Red de enfermedades hepáticas y digestivas (CIBERehd), Instituto de Salud Carlos III, Madrid, Spain.

**Keywords:** oropharyngeal dysphagia, swallowing disorders, risk factors, health determinants, aging

## Abstract

Oropharyngeal dysphagia (OD) is a highly prevalent geriatric syndrome that remains underdiagnosed and undertreated, often resulting in severe nutritional and respiratory complications with poor clinical outcomes. Its risk factors are still not well defined, representing a critical gap in efforts to understand its pathophysiology, prevent the condition and reduce its associated complications. To analyze data from comprehensive studies conducted in a single-center setting to ascertain the major risk factors associated with OD as a geriatric syndrome. A retrospective analysis was conducted, encompassing a review of prior studies, including a cohort of 7,272 older patients with OD at Consorci Sanitari del Maresme, Catalonia, Spain, presented as a narrative review. The study presents data using odds ratios (OR) and p-values from univariate and multivariate analyses to show the association of OD with its main risk factors in different phenotypes of older patients with OD (independently living, acute hospitalized, and with pneumonia, dementia, COVID-19, stroke). Quality of studies was assessed with ROBINS-I-V2. Outcome (risk factors) quality was assessed with GRADE. Thirteen studies (2010–2022) were reviewed. OD exhibited significant associations with 8 main groups of risk factors among older patients from diverse phenotypes. The main risk factors were impaired functionality (OR:2.24-12.7), aging (OR:1.05-5.16) and malnutrition (OR:2.46-5.16). Comorbidities, respiratory disease, neurological impairments, geriatric syndromes and pharmacological treatments were also significantly associated with OD (OR:1.02-15.52). The quality of the included studies and variables was mainly moderate. OD is a geriatric syndrome associated with several risk factors across multiple phenotypes of older patients. These findings highlight the critical need for early identification and targeted prevention of key risk factors for OD to improve clinical outcomes and reduce the burden of this underdiagnosed geriatric syndrome.

## INTRODUCTION

Oropharyngeal dysphagia (OD), or difficulty swallowing, is a highly prevalent condition in older adults and those with neurological or neurodegenerative diseases and is recognized as a geriatric syndrome [1]. It affects an estimated 27% of older persons in the community, 51% of those hospitalized, and 67% of those in nursing home residents [2-5]. Its prevalence is high in post-stroke patients (40-78%) [6] and over 80% in patients with dementia [7,8]. As the world population ages, the incidence of OD is expected to increase in the coming years, resulting in serious health risks and poor clinical outcomes such as malnutrition (MN), dehydration, and aspiration pneumonia [9]. These complications not only lead to increased morbidity and mortality, but also significantly impair quality of life and contribute to social isolation, depression, decreased functional independence, and increased healthcare-associated costs [10-13]. OD meets the criteria for a geriatric syndrome due to its high prevalence in older adults, its association with multiple risk factors—including aging, frailty, sarcopenia, functional impairment, neurogenic conditions, stroke, medications that affect swallowing, and head and neck cancer—and its link to adverse clinical outcomes, such as disability, frailty, functional decline, MN, hospital readmissions, increased hospitalizations, morbidity and mortality [1,8,14,15]. These risk factors have been identified in diverse and heterogeneous studies with smaller numbers of patients from different older phenotypes, different healthcare settings, and even different diagnostic methods for OD [16-20].

Given its multifactorial nature, OD often results from a combination of age-related physiological changes, comorbidities, and lifestyle factors, which complicate both diagnosis and management [5]. The natural aging process induces anatomical changes to the head and neck, as well as alterations in neuronal and muscular mechanisms, leading to a loss of functional reserve that can impact the swallowing process. When these changes occur in healthy and robust older adults without compromising swallowing safety, they are referred to as presbyphagia [21]. However, in the context of OD, these age-related changes in the central and peripheral nervous systems contribute to unsafe swallowing and delayed swallow biomechanics, pharyngeal hypoesthesia with disrupted conduction of pharyngeal sensory inputs, and reduced excitability and delayed cortical motor response [22,23]. Similarly, musculoskeletal changes, including sarcopenia and decreased muscle strength, exacerbate swallowing dysfunction by weakening the muscles responsible for bolus propulsion and airway protection [24,25]. Sarcopenic dysphagia (SD) is an emerging concept introduced in 2012 to describe the link between sarcopenia and OD [26]. SD is a swallowing disorder in older patients caused by sarcopenia that affects the skeletal musculature of the whole body, including the musculature involved in swallowing [27-30]. The prevalence of SD was found to be from 32% to 81% in different patient phenotypes, not only in acute care hospitals but also in long-term care and rehabilitation hospitals and institutionalized older adults [28,31–33]. Finally, neurological conditions prevalent in older adults, such as stroke, Parkinson’s disease, and dementia or the intake of some medications affecting deglutition further reduce efficacy and safety of swallowing [7,34,35].

The interconnected issues of OD, its complications and the contributing factors previously discussed lead to functional decline, prolonged hospital stays for respiratory infections, higher mortality and increased healthcare utilization, which are estimated to cost the U.S. healthcare system between $4 billion and $7 billion annually [5,36]. Studies also show that untreated OD significantly increases hospital stays and healthcare costs, with undetected cases contributing to a 40% increase in inpatient costs [5,9,37].

Early identification of risk factors for OD is critical for prevention and mitigation of its effects. There are three main aspects we have to consider that justify the need to identify OD risk factors: a) The identification of risk factors for OD will contribute to the understanding of its pathophysiology as a geriatric syndrome; b) Identifying risk factors for OD helps clinicians to improve its diagnosis and treatment, to reduce the prevalence of adverse outcomes associated with dysphagia in the aging population, and preserve patients’ quality of life and functionality. In addition, it will help optimize healthcare resources and reduce healthcare costs by decreasing hospitalizations, readmissions, and the need for long-term care [2]. c) Screening for these risk factors might enable healthcare providers to implement preventive measures and multidisciplinary interventions to reduce the incidence and burden of OD on healthcare systems [38-41].

The aim of this review is to identify and characterize the main risk factors associated with OD as a geriatric syndrome in older adults. To this end, we performed a comprehensive analysis of multiple studies conducted in a single-center setting at the Consorci Sanitari del Maresme (CSdM), Catalonia, Spain, using standardized diagnostic protocols and rigorous data management procedures. This uniform approach ensures high diagnostic accuracy, methodological consistency, and strong internal validity of the results. By stratifying the analysis according to the most common clinical phenotypes in the older population, this review provides a detailed, phenotype-specific overview of the primary risk factors associated with OD.

This work offers a unified, systematic, and clinically grounded synthesis of risk factors, contributing a novel and practical framework for understanding OD as a geriatric syndrome. This strategy supports the development of a comprehensive and clinically applicable model for OD, incorporating both general and phenotype-specific risk profiles.

## METHODS

This narrative review performed during 2024-2025 by members from the CSdM-UAB-CIBERehd-IGTP research group on Gastrointestinal Physiology integrates data from thirteen studies conducted by the same interdisciplinary research team including physicians, speech and language pathologists, nutritionists, biologists, pharmacists and engineers at CSdM between 2010 and 2022 [3,7,37,42–51]. CSdM is a public health consortium located in the Maresme Area in Catalonia, Spain that includes primary care, acute and sub-acute hospitalization (Mataró University Hospital), rehabilitation and nursing homes for a population of 278,954 individuals, and mental health and addictions for a population of 445,720 people (2023).

### Study Inclusion Criteria

The thirteen studies evaluating OD risk factors were chosen based on their shared focus and consistent execution, ensuring alignment in both research aims and methodology. All studies were carried out at Mataró University Hospital by the same research team, which provided a consistent setting and personnel and standardized methodologies, making the studies highly comparable. The research methods employed across these studies were intentionally similar, facilitating a cohesive analysis and ensuring that the data could be cross-referenced effectively. Finally, each study was overseen by healthcare professionals and clinical researchers with substantial expertise in the field, which guaranteed the methodological rigor of the research and reliability of the results.

The thirteen studies included in this review collectively examined various aspects related to OD and its complications, focusing on older populations (>65 years) in diverse clinical contexts and phenotypes, including post-stroke care, COVID-19-related hospitalization, community-dwelling older people, dementia care, and geriatric and internal medicine wards. Homogeneous and highly accurate assessment tools were employed across the studies, with the Volume-Viscosity Swallow Test (V-VST) [52,53] prominently used in twelve studies to assess swallowing safety and efficacy. In addition to the V-VST, two studies employed other screening tools, the *Eating Assessment Tool* (EAT-10) and the water swallow test (WST) [54,55]. Two studies that utilized the V-VST also incorporated instrumental assessment by using videofluoroscopy (VFS).

### Study variables

The studies consistently evaluated a comprehensive set of variables to identify potential risk factors for OD or its impairments in each phenotype, encompassing:

1. Demographic factors: Age (both continuous and categorical), gender, and population setting (home-dwelling, hospitalized or nursing home).
2. Functional capacity: Assessed using the Barthel Index [56] and modified Rankin Scale (mRS) [57] to quantify independence and functional limitations.
3. Nutritional status: Measured with tools such as the Mini Nutritional Assessment (MNA) [58] and the Global Leadership Initiative on Malnutrition (GLIM) [59] and laboratory parameters like serum albumin, protein and cholesterol levels.
4. Geriatric Syndromes: Included delirium, frailty, urinary and fecal incontinence, immobility, pressure ulcers, and dementia [14].
5. Comorbidities: Included chronic diseases such as cardiovascular, renal, and cerebrovascular conditions. The Charlson Comorbidity Index [60] was used to evaluate overall comorbidity burden. Acute conditions, such as pneumonia and urinary tract infections, were also included in this category.
6. Neurological conditions: Included stroke (severity, lesion volume, and location), as well as chronic neurological symptoms, neurodegenerative and cerebro-vascular diseases and different types of impaired cognitive function and dementia.
7. Medication: Use of sedatives, antipsychotics, antidepressants, neuroleptics, and other compounds which may affect swallowing function [47].
8. Respiratory and swallowing parameters: Episodes of lower respiratory tract infections (LRTI), bronchoaspiration, pneumonia severity (Fine index) [61].
9. Hospital-related outcomes: Duration of hospital stay, institutionalization (e.g., discharge to a nursing home) and readmissions.

By systematically evaluating these variables, the studies established a consistent methodological framework that enabled comparisons across different clinical populations and settings at the CSdM. This comprehensive approach ensured a robust foundation for identifying the main risk factors associated with OD and the specific ones related to 5 main phenotypes of older patients with OD: a) independently living, b) hospitalized in general hospitals with acute diseases, c) pneumonia, d) acute and chronic post stroke OD, e) patients with dementia f) and those affected by COVID-19 during the recent pandemic.

### Evidence Quality Evaluation

To ensure a robust assessment of the risk of bias across non-randomized studies included in this review, we used the updated version of the ROBINS-I tool (Risk Of Bias In Non-randomized Studies – of Interventions, Version 2) [62]. This tool, launched in November 2016, is specifically designed to evaluate the risk of bias in cohort studies and other non-randomized designs by comparing their results to a hypothetical, well-conducted randomized trial (the "target trial").

The ROBINS-I V2 framework assesses seven critical domains (D) of bias: D1) Bias due to confounding; D2) Bias in the classification of interventions; D3) Bias due to selection of participants into the study; D4) Bias due to deviations from intended interventions; D5) Bias due to missing data; D6) Bias in measurement of outcomes, and D7) Bias in the selection of the reported result.

Each domain was assessed using structured signaling questions, and judgments were made on the extent of bias present, categorized as low, moderate, serious, or critical by two independent reviewers from the research team, neither of whom had participated in any of the included studies (LG, JIC-M). This rigorous approach allowed us to systematically evaluate and present the strengths and limitations of evidence derived from non-randomized studies.

### Outcome (Risk Factor) Quality Evaluation

This study used the Grading of Recommendations, Assessment, Development, and Evaluations (GRADE) framework to systematically evaluate the quality of evidence for risk factors associated with OD in older adults [63]. The GRADE approach offers a transparent and structured process for assessing evidence quality across five key domains: 1) Risk of Bias; 2) Inconsistencies; 3) Indirectness; 4) Imprecision; and 5) Publication Bias.

Each study was assigned an overall quality rating for the identified risk factors, ranging from very low to high, by two independent reviewers from the research team neither of whom had been involved in any of the included studies The quality ratings informed the synthesis and interpretation of evidence, ensuring a rigorous evaluation of the risk factors for OD in older adults. Detailed assessments and corresponding ratings are presented in the accompanying tables, which form the foundation for subsequent discussions on clinical implications.

### Statistical Analysis

Studies included in this review employed diverse and robust statistical methodologies to investigate the prevalence, risk factors, and impact of OD. Given the heterogeneity of study designs and outcomes, a narrative synthesis was performed. We systematically extracted and synthesized data on the prevalence of OD, as well as associations between OD and potential risk factors. When available, we extracted odds ratios (ORs), confidence intervals (CIs), and p-values from univariate and multivariate analyses reported in the original studies.

We classified the strength of associations using standard OR thresholds: weak (1.1–1.9), moderate (2.0–4.9), and strong (≥5.0) associations [64]. Due to differences in populations, settings, and outcome definitions, no meta-analysis was performed. Instead, we compared effect sizes across studies and summarized findings according to main clinical phenotypes. No additional statistical analysis was performed beyond synthesis and qualitative comparison of results reported in the included studies.

## RESULTS

### Primary findings

Thirteen studies conducted between 2010 and 2022 were included in this review, comprising a cumulative cohort of 7,272 individuals >65 years evaluated for OD across diverse clinical settings at CSdM. These studies assessed a wide range of variables related to OD risk factors, employing validated clinical and diagnostic tools such as the V-VST and videofluoroscopy.

The thirteen studies included in this review employed diverse and rigorous statistical methodologies to investigate the prevalence, risk factors, and outcomes associated with OD. Most studies applied both univariate and multivariate techniques to explore relationships between OD and clinical, demographic, and functional variables.

Descriptive analyses were used to report the prevalence and basic distributions of OD and its associated variables. Univariate analyses included chi-square and Fisher’s exact tests for categorical variables, and t-tests or Mann–Whitney U tests for continuous variables, to identify preliminary associations. Multivariate analyses were most commonly conducted using logistic regression to identify independent predictors of OD, adjusting for potential confounders. Some studies also used Cox proportional hazards models and Kaplan–Meier survival analyses to assess time-dependent outcomes such as mortality, hospital readmission, or institutionalization.

Brief summaries of the thirteen studies are presented below in chronological order. Table 1 shows a summary of the main risk factors across the studies and Table 2, the comparison of the risk factors among the main phenotypes included.

Study 1. Cabré M *et al.* [43] assessed the prevalence and prognostic significance of OD using the adapted water swallow test (aWST) on 134 older patients (mean age 84.5 ± 6.8 years) admitted with pneumonia and compared patients with OD with those without (no-OD). Dysphagia prevalence was 55.2% and was associated with significantly higher 30-day mortality (OR = 3.28) and 1-year mortality (OR = 3.42). Functional status and MN emerged as stronger predictors of mortality than comorbidities. Several factors were found to be significantly associated with OD compared to the no-OD group. These included advanced age (>85 years), poor functional status (low Barthel Index scores both pre-admission and during admission), MN or at-risk nutritional status (MNA <17 or 17–23.5), fecal and urinary incontinence, pressure ulcers, immobility, and prior falls. Additionally, living in or being discharged to a nursing home, high Fine’s pneumonia severity scores, new or increased confusion, and the use of antipsychotic medication were significantly more frequent in the OD group. In contrast, gender and chronic diseases such as heart failure and diabetes were not significantly associated with OD.

**Table 1.** Summary of the main risk factors across the 13 clinical studies included in the review.

Risk factor	Phenotype	Outcome	Diagnosis tool	Analysis	P-value	OR	Results tendency	Ref.
Age (>85 years)	Pneumonia	SI	aWST	Univariate	0.044	NR	↑ Age (>85y) ↓ SI	[43]
Antipsychotics	Pneumonia	SI	aWST	Univariate	<0.001	NR	↑ Use antipsychotic medications ↑ SI	[43]
Barthel Index at admission	Pneumonia	SI	aWST	Univariate	<0.001	NR	↓ Functional capacity at admission ↑ SI	[43]
Barthel Index pre-admission	Pneumonia	SI	aWST	Univariate	<0.001	NR	↓ Functional capacity before admission > SI	[43]
Charlson Comorbidity Index	Pneumonia	SI	aWST	Univariate	<0.001	NR	↑ Comorbidities ↑ SI	[43]
Fecal incontinence	Pneumonia	SI	aWST	Univariate	<0.001	NR	↑ Fecal incontinence ↑ SI	[43]
Immobility	Pneumonia	SI	aWST	Univariate	<0.001	NR	↑ Rates of immobility ↑ SI	[43]
Living in a nursing home	Pneumonia	SI	aWST	Univariate	0.007	NR	↑ Living in nursing home ↑ SI	[43]
MN (MNA < 17)	Pneumonia	SI	aWST	Multivariate	0.004	NR	↑ MN ↑ SI	[43]
New or increased confusion	Pneumonia	SI	aWST	Univariate	0.002	NR	↑ New or worsening confusion ↑ SI	[43]
Pneumonia severity (Fine score)	Pneumonia	SI	aWST	Univariate	0.006	NR	↑ Pneumonia severity scores ↑ SI	[43]
Pressure ulcers	Pneumonia	SI	aWST	Univariate	0.005	NR	↑ Pressure ulcers ↑ SI	[43]
Prior falls	Pneumonia	SI	aWST	Univariate	<0.001	NR	↑ History of falls ↑ SI	[43]
To nursing home (discharge)	Pneumonia	SI	aWST	Univariate	0.01	NR	↓ Discharge at home ↑ SI	[43]
Urinary incontinence	Pneumonia	SI	aWST	Univariate	<0.001	NR	↑ Urinary incontinence ↑ SI	[43]
Barthel Index at admission	Acute disease	SI	VFS	Univariate	0.002	NR	↓ Functional capacity at admission ↑ SI	[48]
Barthel Index pre-admission	Acute disease	SI	VFS	Univariate	0.025	NR	↓ Functional capacity at pre-admission ↑ SI	[48]
Age (≥ 80)	IL older adults	EI	V-VST	Multivariate	0.02	2.23 (1.14–4.35)	↑ Age (≥ 80y) ↑ EI	[3]
Barthel Index (<100)	IL older adults	OD	V-VST	Multivariate	<0.001	3.35 (1.68–6.70)	↓ Barthel ↑ OD	[3]
Barthel Index (<100)	IL older adults	EI	V-VST	Multivariate	0.03	2.24 (1.09–4.58)	↓ Barthel ↑ EI	[3]
Barthel Index (<100)	IL older adults	SI	V-VST	Multivariate	0.01	2.76 (1.23–6.17)	↓ Barthel ↑ IS	[3]
Risk of MN	IL older adults	EI	V-VST	Multivariate	0.03	2.46 (1.10–5.46)	↑ MN ↑ EI	[3]
Age	IL older adults	OD	V-VST	Univariate	<0.001	NR	↑ Age↑ OD	[50]
Barthel Score	IL older adults	OD	V-VST	Univariate	<0.001	NR	↓ Functional capacity ↑ OD	[50]
Benzodiazepines	IL older adults	OD	V-VST	Univariate	0.046	NR	↑ Benzodiazepines use ↑ OD	[50]
Hand grip (Men)	IL older adults	OD	V-VST	Univariate	0.009	NR	↓ Hand grip ↑ OD	[50]
Walking speed	IL older adults	OD	V-VST	Univariate	0.044	NR	↓ Walking speed ↑ OD	[50]
Age (>85 years)	Pneumonia	OD	V-VST	Univariate	<0.001	NR	↑ Age ↑ OD	[44]
Albumin levels	Pneumonia	OD	V-VST	Univariate	<0.001	NR	↓ Albumin levels (poorer nutritional status) ↑ OD	[44]
Antidepressant drugs	Pneumonia	OD	V-VST	Univariate	<0.001	NR	↑ Use antidepressants ↑ OD	[44]
Antipsychotic drugs	Pneumonia	OD	V-VST	Univariate	<0.001	NR	↑ Use antipsychotic medications ↑ OD	[44]
BMI (Body Mass Index)	Pneumonia	OD	V-VST	Univariate	<0.001	NR	↓ BMI (poorer nutritional status) ↑ OD	[44]
Barthel Index at admission	Pneumonia	OD	V-VST	Univariate	<0.001	NR	↓ Functional status at admission ↑ OD	[44]
Barthel Index at discharge	Pneumonia	OD	V-VST	Univariate	<0.001	NR	↓ Functional recovery at discharge ↑ OD	[44]
Barthel Index pre-admission	Pneumonia	OD	V-VST	Univariate	<0.001	NR	↓ Functional capacity before admission ↑ OD	[44]
Benzodiazepines	Pneumonia	OD	V-VST	Univariate	0,003	NR	↑ Use benzodiazepines ↑ OD	[44]
Cerebrovascular disease	Pneumonia	OD	V-VST	Univariate	<0.001	NR	↑ Cerebrovascular disease ↑ OD	[44]
Charlson Comorbidity Index	Pneumonia	OD	V-VST	Univariate	<0.001	NR	↑ Comorbidities ↑ OD	[44]
Cholesterol levels	Pneumonia	OD	V-VST	Univariate	0,011	NR	↓ Cholesterol levels (↓ overall health) ↑ OD	[44]
Delirium	Pneumonia	OD	V-VST	Univariate	<0.001	NR	↑ Delirium ↑ OD	[44]
Dementia	Pneumonia	OD	V-VST	Univariate	<0.001	NR	↑ Dementia ↑ OD	[44]
Fecal incontinence	Pneumonia	OD	V-VST	Univariate	<0.001	NR	↑ Fecal incontinence ↑ OD	[44]
Hand grip strength (men)	Pneumonia	OD	V-VST	Univariate	<0.001	NR	↓ Hand grip strength ↑ OD	[44]
Hand grip strength (women)	Pneumonia	OD	V-VST	Univariate	<0.001	NR	↓ Hand grip strength ↑ OD	[44]
Immobility	Pneumonia	OD	V-VST	Univariate	<0.001	NR	↑ Immobility ↑ OD	[44]
Ischemic heart disease	Pneumonia	OD	V-VST	Univariate	<0.001	NR	↑ Ischemic heart disease ↑ OD	[44]
Living in a nursing home	Pneumonia	OD	V-VST	Univariate	<0.001	NR	↑ Living in nursing homes ↑ OD	[44]
MN (MNA < 17)	Pneumonia	OD	V-VST	Multivariate	<0.001	NR	↑ MN ↑ OD	[44]
Pressure ulcers	Pneumonia	OD	V-VST	Univariate	<0.001	NR	↑ Pressure ulcers ↑ OD	[44]
Previous falls	Pneumonia	OD	V-VST	Univariate	0.001	NR	↑ History of falls ↑ OD	[44]
Urinary incontinence	Pneumonia	OD	V-VST	Univariate	<0.001	NR	↑ Urinary incontinence ↑ OD	[44]
Acute renal failure	Acute disease	OD	V-VST	Univariate	0.002	1.73 (1.21–2.47)	↑ Acute renal failure ↑ OD	[45]
Age (>85 years)	Acute disease	OD	V-VST	Univariate	<0.001	1.6 (1.3–1.9)	↑ Age↑ OD	[45]
Antidepressants	Acute disease	OD	V-VST	Univariate	0.001	1.46 (1.16–1.84)	↑ Antidepressant use ↑ OD	[45]
Barthel Index pre-admission (<40)	Acute disease	OD	V-VST	Univariate	<0.001	9.57 (7.25–12.65)	↓ Functional capacity (Barthel Index <40) ↑ OD	[45]
Barthel Index during-admission (<40)	Acute disease	OD	V-VST	Univariate	<0.001	9.71 (7.23 -13.04)	↓ Functional capacity (Barthel Index <40) ↑ OD	[45]
Barthel Index at discharge (<40)	Acute disease	OD	V-VST	Univariate	<0.001	12.7 (6.5–16.8)	↓ Functional capacity (Barthel Index <40) ↑ OD	[45]
Cerebrovascular diseases	Acute disease	OD	V-VST	Univariate	<0.001	1.94 (1.53–2.46)	↑ Prevalence of cerebrovascular disease ↑ OD	[45]
Charlson Comorbidity Index	Acute disease	OD	V-VST	Univariate	<0.001	1.16 (1.09–1.24)	↑ Charlson Index ↑ OD	[45]
Chronic renal failure	Acute disease	OD	V-VST	Univariate	0.003	1.44 (1.12–1.84)	↑ Prevalence of chronic renal failure ↑ OD	[45]
Dementia	Acute disease	OD	V-VST	Univariate	<0.001	4.69 (3.73–5.90)	↑ Prevalence of dementia ↑ OD	[45]
Faecal incontinence	Acute disease	OD	V-VST	Univariate	<0.001	5.76 (4.60–7.22)	↑ Prevalence of fecal incontinence ↑ OD	[45]
Immobility syndrome	Acute disease	OD	V-VST	Univariate	<0.001	5.65 (4.54–7.03)	↑ Immobility ↑ OD	[45]
Ischemic heart disease	Acute disease	OD	V-VST	Univariate	0.002	0.67 (0.52–0.86)	↓ Ischemic heart disease ↑ OD	[45]
Neoplasia	Acute disease	OD	V-VST	Univariate	0.029	0.71 (0.53–0.96)	↓ Neoplasia ↑ OD	[45]
Neuroleptics	Acute disease	OD	V-VST	Univariate	<0.001	3.35 (2.48–4.53)	↑ Neuroleptic use ↑ OD	[45]
Pressure ulcers	Acute disease	OD	V-VST	Univariate	<0.001	5.92 (3.73–9.38)	↑ Pressure ulcers ↑ OD	[45]
Previous falls	Acute disease	OD	V-VST	Univariate	0.02	1.29 (1.03 -1.61)	↑ Previous falls ↑ OD	[45]
Sedatives	Acute disease	OD	V-VST	Univariate	0.005	1.33 (1.09–1.63)	↑ Use sedatives ↑ OD	[45]
Urinary incontinence	Acute disease	OD	V-VST	Univariate	<0.001	4.00 (3.23–4.96)	↑ Urinary incontinence ↑ OD	[45]
Age (>70 years)	Acute disease	OD	V-VST	Univariate	<0.001	1.05 (1.03–1.07)	↑ Age ↑ OD	[47]
Albumin	Acute disease	OD	V-VST	Univariate	<0.001	1.90 (1.25–2.31)	↓ Album↑ OD	[47]
Antidepressant	Acute disease	IS	V-VST	Univariate	0.011	1.44 (1.09–1.91)	↑ Antidepressant ↑ OD	[47]
Antipsychotic	Acute disease	OD	V-VST	Univariate	0.002	1.93 (1.27–2.94)	↑ Antipsychotic use ↑ OD	[47]
Barthel Index pre-admission (<40)	Acute disease	OD	V-VST	Univariate	<0.001	5.10 (4.31-8.34)	↓ Functional capacity (Barthel Index <40) ↑ OD	[47]
BMI	Acute disease	OD	V-VST	Univariate	<0.001	1.47 (1.03–2.09)	↓ BMI ↑ OD	[47]
Cerebrovascular disease	Acute disease	OD	V-VST	Univariate	<0.001	1.88 (1.37–2.57))	↑ Cerebrovascular disease ↑ OD	[47]
Charlson Index	Acute disease	OD	V-VST	Univariate	<0.001	1.16 (1.08–1.24)	↑ Charlson Index ↑ OD	[47]
Confusion	Acute disease	OD	V-VST	Univariate	<0.001	1.91 (1.45–2.52)	↑ Confusion ↑ OD	[47]
Dementia	Acute disease	OD	V-VST	Univariate	< 0.001	5.93 (4.33–8.13)	↑ Dementia ↑ OD	[47]
Drugs against dementia	Acute disease	IS	V-VST	Univariate	0.001	2.73 (1.47–5.07)	↑ Drugs against dementia use ↑ OD	[47]
Drug for Nervous system	Acute disease	OD	V-VST	Univariate	0.002	1.62 (1.17–2.24)	↑ Drug for Nervous system use ↑OD	[47]
Handgrip (kg) Women	Acute disease	OD	V-VST	Univariate	<0.001	0.84 (0.81- 0.88)	↓ Hand grip ↑ OD	[47]
Handgrip (kg) Men	Acute disease	OD	V-VST	Univariate	<0.001	0.89 (0.86–0.92)	↓ Hand grip ↑ OD	[47]
Hypertension	Acute disease	OD	V-VST	Univariate	<0.001	0.22 (0.48–0.79)	↓ Hypertension ↑ OD	[47]
Neurodegenerative diseases	Acute disease	OD	V-VST	Univariate	< 0.001	2.25 (1.61–3.16)	↑ Neurodegenerative disease ↑ OD	[47]
Renal failure	Acute disease	OD	V-VST	Univariate	0.002	1.75 (1.24–2.48)	↑ Renal failure ↑ OD	[47]
Respiratory diseases	Acute disease	OD	V-VST	Univariate	0.049	0.76 (0.57–1.00)	↓ Respiratory disease ↑ OD	[47]
Underweight (<24)	Acute disease	OD	V-VST	Univariate	<0.001	1.47 (1.03–2.09)	↑ Underweight ↑ OD	[47]
Age	Acute disease	OD	VFS + V-VST	Univariate	0.005	NR	↑ Age ↑ OD	[46]
Barthel index	Acute disease	OD	VFS + V-VST	Univariate	0.003	NR	↓ Functional status at hospital admission ↑ OD	[46]
Charlson Comorbidity Index	Acute disease	OD	VFS + V-VST	Univariate	0.001	NR	↑ Charlson Index ↑ OD	[46]
Handgrip strength	Acute disease	OD	VFS + V-VST	Univariate	0.001	NR	↓ Handgrip ↑ OD	[46]
MN	Acute disease	OD	VFS + V-VST	Univariate	0.006	NR	↑ MN↑ OD	[46]
Polypharmacy	Acute disease	OD	VFS + V-VST	Univariate	0.007	NR	↑ Use polypharmacy medications ↑ OD	[46]
Previous Stroke	Acute disease	OD	VFS + V-VST	Univariate	<0.001	NR	↑ Stroke ↑ OD	[46]
Age (per year)	Stroke	OD	V-VST	Multivariate	<0.001	1.05 (1.02–1.08)	↑ Age ↑ OD	[49]
Charlson Index	Stroke	OD	V-VST	Univariate	<0.001	1.22 (1.08-1.37)	↑ Charlson Index ↑ OD	[49]
Functional status (mRS >1)	Stroke	OD	V-VST	Univariate	<0.001	3.00 (1.58–5.68)	↑ Functional outcomes previous the stroke ↑ OD	[49]
Heart disease	Stroke	OD	V-VST	Univariate	0.013	1.78 (1.15-2.79)	↑ Heart disease ↑ OD	[49]
Hypertension	Stroke	OD	V-VST	Univariate	<0.001	1.74 (1.06-2.88)	↑ Hypertension ↑ OD	[49]
Infarct	Stroke	OD	V-VST	Univariate	0.014	2.20 (1.16-4.16)	↑ Infarct ↑ OD	[49]
Marital Status	Stroke	OD	V-VST	Univariate	<.001	2.24 (1.45 - 3.48)	↑ Windowed ↑ OD	[49]
Major cardioembolic	Stroke	OD	V-VST	Univariate	0.012	2.00 (1.19-3.37)	↑ Cardioembolic event ↑ OD	[49]
Nursing Home (living)	Stroke	OD	V-VST	Univariate	<.001	7.53 (2.17-26.14)	↑ Nursing home ↑ OD	[49]
NIHSS (>6)	Stroke	OD	V-VST	Multivariate	0.002	3.52 (1.57–7.87)	↑ Stroke severity (NIHSS >6) ↑ OD	[49]
Oxford Stroke Classification (TACI)	Stroke	OD	V-VST	Univariate	<.001	15.52 (5.41-44.56)	↑ Anterior circulation involvement ↑ OD	[49]
Previous stroke	Stroke	OD	V-VST	Univariate	0.038	1.73 (1.04-2.89)	↑ In patients with previous stroke ↑ OD	[49]
Prolonged hospital stay	Stroke	OD	V-VST	Univariate	0.049	β = 0.938	↑ Hospital stays ↑ OD	[49]
Sex	Stroke	OD	V-VST	Univariate	0.041	1.52 (1.02 - 2.26)	Women ↑ OD	[49]
Stroke lesion volume (per cc)	Stroke	OD	V-VST	Multivariate	0.004	1.02 (1.01–1.03)	↑ Stroke lesion volume ↑ OD	[49]
Territorial infarction findings	Stroke	OD	V-VST	Univariate	0.006	2.20 (1.19-3.37)	↑ Territorial infarction findings ↑ OD	[49]
Age (per year)	Stroke	SI	V-VST	Univariate	0.009	NR	↑ Age ↑ SI	[42]
No previous heart disease	Stroke	SI	V-VST	Univariate	0.015	3.9 (1.3–11.9)	↑ Previous heart disease ↑ SI	[42]
No previous heart disease	Stroke	EI	V-VST	Univariate	0.019	4.6 (1.2–17.3)	↑ Previous heart disease ↑ EI	[42]
Pre-stroke Ranking score	Stroke	EI	V-VST	Univariate	0.045	NR	↑ Pre-stroke Ranking score ↑ SI	[42]
Barthel Index on discharge	Stroke	EI	V-VST	Univariate	0.046	NR	↑ Barthel Score ↑ EI	[42]
MN (MNA-sf)	Stroke	EI	V-VST	Univariate	0.033	NR	↑ MNA-sf ↑ EI	[42]
Age (per year)	Dementia	OD	V-VST	Univariate	0.007	NR	↑ Age ↑ OD	[7]
Barthel (Admission)	Dementia	OD	V-VST	Univariate	< 0.0001	NR	↓ Functional capacity ↑ OD	[7]
Dementia severity (GDS)	Dementia	OD	V-VST	Univariate	< 0.0001	NR	↑ Dementia severity (GDS higher) ↑ OD	[7]
Dementia severity (Fast)	Dementia	OD	V-VST	Univariate	0.007	NR	↑ dementia severity (FAST) ↑ OD	[7]
MN (MNA-sf)	Dementia	OD	V-VST	Univariate	0.014	NR	↑ MN ↑ OD	[7]
Age	COVID-19	OD	V-VST	Univariate	<0.0001	NR	↑ Age ↑ OD	[37]
Albumin levels	COVID-19	OD	V-VST	Univariate	0.025	NR	↓ Albumin level ↑ OD	[37]
Barthel Index	COVID-19	OD	V-VST	Multivariate	0.008	0.92 (0.87-0.98)	↓ Pre-admission functionality ↑ OD	[37]
Charlson Comorbidity Index	COVID-19	OD	V-VST	Multivariate	0.040	1.49 (1.02-2.18)	↑ Comorbidity index ↑ OD	[37]
Confusion	COVID-19	OD	V-VST	Univariate	0.0001	NR	↑ Confusion ↑ OD	[37]
Delirium	COVID-19	OD	V-VST	Multivariate	0.013	10.97 (1.64-73.31)	↑ Delirium ↑ OD	[37]
Mean weight	COVID-19	OD	V-VST	Univariate	<0.0001	NR	↓ Mean weigh ↑ OD	[37]
Neurological Symptoms:	COVID-19	OD	V-VST	Univariate	0.0001	NR	↑ Neurological symptoms ↑ OD	[37]
Protein level	COVID-19	OD	V-VST	Univariate	0.025	NR	↓ Protein Level ↑ OD	[37]
Age (per year)	COVID-19	OD	V-VST + EAT-10	Multivariate	<0.001	5.16 (2.44–10.90)	↑ Age ↑ OD	[51]
Barthel Index (Severe Dependency)	COVID-19	OD	V-VST + EAT-10	Multivariate	<0.001	3.62 (2.12–6.18)	↑ Functional dependency (Barthel <40) ↑ OD	[51]
Delirium	COVID-19	OD	V-VST + EAT-10	Multivariate	<0.001	7.09 (2.84–17.69)	↑ Delirium ↑ OD	[51]
MN (NRS-2002 ≥ 3)	COVID-19	OD	V-VST + EAT-10	Multivariate	<0.001	5.16 (2.44–10.90)	↑ MN ↑ OD	[51]

Symbols and abbreviations: ↑: higher; ↓: lower; aWST: adapted water swallow test; BMI: body mass index; CAP: community-acquired pneumonia; EAT-10: eating assessment tool; EI: efficacy impairment; GDS: global deterioration scale; IL: independently-living; LRTI: lower respiratory tract infection; MNA: mini-nutritional assessment form; MN: malnutrition; mRS: modified Rankin scale; NDD: neurodegenerative disease; NIHSS: National Institutes of Health Stroke Scale; NR: not reported; OD: oropharyngeal dysphagia; OR: ODDs ratio; SI: safety impairment; SS: safe swallow; VFS: videofluoroscopy; V-VST: volume-viscosity swallowing test.

Study 2. Rofes et al. [48] compared the patho-physiology of OD in 45 frail older patients (mean age 81.5 ± 1.1 years) and 12 healthy volunteers (mean age 40.2 ± 2.5 years) using VFS. Impaired swallowing safety was significantly linked to reduced functional capacity, with lower Barthel Index scores both before admission (p = 0.025) and at the time of admission (p = 0.002).

Study 3. Serra-Prat M *et al.* [3] investigated the prevalence of OD and its risk factors in a population-based cohort study on 254 independently living older adults in the city of Mataró, Catalonia, Spain (mean age 78.2 ± 5.6 years). Using the V-VST, OD prevalence was detected in 27.2% of participants. A significant risk factor for impaired swallowing safety and efficacy was low functional capacity (Barthel Index <100, OR = 3.35 for OD; OR = 2.76 for impaired safety; OR = 2.24 for impaired efficacy). Impaired swallowing efficacy was also significantly predicted by advanced age (≥80 years, OR = 2.23) and being malnourished or at risk of MN (MNA <24, OR = 2.46). Benzodiazepine use, neurodegenerative diseases, depression, and gender were not significant predictors of dysphagia.

**Table 2.** Most relevant and frequent risk factors classified according to patients’ phenotype.

RISK FACTOR		Independent living (n=254)		Acute (n=2.818)				Pneumonia (n=2.493)		Stroke (n=642)		Dementia (n=255)	COVID (n=810)		Summary
		Serra-Prat 2011 [3]	Serra-Prat 2012 [50]	Carrión 2 017 [46]	Rofes 2010 [48]	Carrión 2015	Miaron 2016 [47]	Cabré 2010 [43]	Cabré 2014 [44]	Rofes 2018 [49]	Arreola 2019 [42]	Espinosa-Val MC 2020 [7]	Martin-Martinez A 2021 [23]	Viñas P 2022 [51]	-
**Reduced functional capacity**	Analysis	**M**	U	U	U	U	U	U	U	U	U	U	**M**	**M**	**U/M**
	OR	3.35	-	-	-	9.71	5.10	-	-	3.00	-	-	0.92	3.62	9.71-0.92
	P-value	<0.001	<0.001	0.003	0.002	<0.001	<0.001	<0.001	<0.001	<0.001	0.046	< 0.0001	0.008	<0.001	<0.05
**Advanced age**	Analysis	**M**	U	U		U	U	U	U	**M**	U	U	U	**M**	**U/M**
	OR	2.23	-	-		1.6	1.05	-	-	1.05	-	-	-	5.16	5.16-1.05
	P-value	0.02	<0.001	0.005		<0.001	<0.001	0.044	<0.001	<0.001	0.009	0.007	<0.0001	<0.001	<0.05
**Malnutrition**	Analysis	**M**		U			U	**M**	**M**		U	U	U	**M**	**U/M**
	OR	2.46		-			1.90	-	-		-	-	-	5.16	5.16-2.46
	P-value	0.03		0.006			♢ <0.001	0.004	<0.001		0.033	0.014	♢♦ 0.025	<0.001	<0.05
**Comorbidity**	Analysis					U	U	U	U	U			**M**		**U/M**
	OR					1.6	1.16	-	-	1.22			1.49		1.6-1.22
	P-value					<0.001	<0.001	<0.001	<0.001	<0.001			0.040		<0.05
**Delirium**	Analysis								U				**M**	**M**	**U/M**
	OR								-				10.97	7.09	10.97-7.09
	P-value								<0.001				0.0001	<0.001	<0.01

♢Malnutrition parameter used: Albumin level; ♦ Malnutrition parameter used: Protein level; U: univariate analysis; M: multivariate analysis. The only other significant risk factors with a multivariate analysis were found in stroke phenotype: NIHSS (>6): 0.002; OR: 3.52 (1.57–7.87); Stroke lesion volume (per cc): 0.004; OR: 1.02 (1.01–1.03)

Study 4. Serra-Prat M *et al.* [50] investigated the role of OD in MN risk in a subsequent analysis of the same cohort from Study 3 [50] after 1 year follow-up. Dysphagia prevalence was determined to be 23.0% using the V-VST. OD was significantly associated with advanced age and low functional capacity (Barthel Index <100), while low hand grip strength and slow walking speed were also significantly linked to OD. In contrast, the use of benzodiazepines was not found to be significantly associated with OD. Gender and baseline nutritional status were not predictive of OD. The study demonstrated that OD significantly increased the risk of MN and low tract respiratory infections at 1 year follow up.

Study 5. Cabre M *et al.* [44] investigated OD as a risk factor for pneumonia-related hospital readmissions in an observational cohort study on 2,359 older patients (mean age 84.9 ± 6.2 years) discharged from an acute geriatric unit (AGU) at CSdM. The prevalence of OD detected with V-VST was 47.5% and was significantly associated with older age (>85 years old), comorbidity (Charlson Comorbidity Index), poor functional status (low Barthel Index scores on admission, pre-admission, and discharge), MN (MNA <17), BMI, and albumin levels. Other significant associations included dementia, ischemic heart disease, cerebrovascular disease, urinary and fecal incontinence, pressure ulcers, immobility, poor hand grip strength (in both men and women), prior falls, cholesterol levels, the use of antidepressant and antipsychotic drugs, living in a nursing home, and the presence of delirium.

Study 6. Carrión S *et al.* [45] examined the relationship between OD, MN, and clinical outcomes in an observational study using the V-VST on 1,662 older adults (mean age 85.1 ± 6.3 years) hospitalized with acute illnesses. OD prevalence was 47.4%, and significant risk factors included advanced age (>85 years, OR 1.73), poor functional capacity (Barthel Index <40, OR 9.57), and a higher comorbidity burden (Charlson Comorbidity Index, OR 1.16). Geriatric syndromes, such as dementia (OR 4.69), immobility syndrome (OR 5.65), fecal incontinence (OR 5.76), urinary incontinence (OR 4.00), and pressure ulcers (OR 5.92), were significantly associated with OD. Chronic renal failure (OR 1.44) and acute renal failure (OR 1.73) were also notable contributors. Polypharmacy and the use of sedatives (OR 1.33), antidepressants (OR 1.46), and neuroleptics (OR 3.35) further increased OD risk. Cerebrovascular diseases (OR 1.94) showed a strong association with OD. Previous falls (OR 3.02) were also significantly linked to OD, while ischemic heart disease (OR 0.67) and neoplasia (OR 0.71) exhibited inverse associations.

Study 7. Miarons M *et al.* [47] examined the association between chronic exposure to medications and OD in a retrospective cross-sectional study on 966 older patients admitted to the AGU (mean age 85.3 ± 6.4). Patients were divided into two groups, patients with OD and patients without OD. Older age (>70; OR 1.05) and lower Barthel scores for functional capacity (<40; OR 5.10) were significantly associated with OD. Cerebrovascular disease (1.88), chronic renal failure comorbidities (OR 1.75) and neurodegenerative diseases (OR 2.25) were also statistically associated with OD as well as higher Charlson Comorbidity Index scores (OR 1.16). Almost all the geriatric syndromes, handgrip (women OR 0.84; men OR 0.89), dementia (OR 5.93) and confusion (OR 1.91), with the exception of depression, were also risk factors for OD. Hypertension (OR 0.22) and respiratory diseases (OR 0.76) were more frequent in the group of patients without OD and polypharmacy was similar among patients with and without OD. In addition, lower BMI scores (OR 1.47) and albumin levels (OR 1.90) were also closely associated with OD.

Study 8. Carrión S *et al.* [46] investigated the nutritional status of older patients with OD in this observational cross-sectional study in chronic versus acute clinical settings. The study included 95 patients with OD due to chronic neurological conditions or aging (mean age 79.1 ± 0.6 years), 23 patients with OD and acute community-acquired pneumonia (CAP) (mean age 83.1 ± 1.6 years), and 15 controls without OD (mean age 76.0 ± 0.54 years). Significant findings were a high prevalence of MN (MNA < 23.5) and polypharmacy (p = 0.007) in patients with OD. Reduced hand grip strength (p = 0.001), advanced age (p = 0.005), poor functional capacity (Barthel Index < 40, p = 0.003), and comorbidity (Charlson Comorbidity Index, p = 0.001) were also significant risk factors for OD. Previous stroke was also associated with chronic OD (p <0.001).

Study 9. Rofes S *et al.* [48] evaluated OD prevalence and its risk factors in this longitudinal cohort study on 395 patients with acute stroke (mean age 73.2 ± 13.1 years). OD was assessed using the V-VST and VFS, revealing a prevalence of 45.06%. Functional status, mortality, respiratory infections, and readmissions were evaluated at 3 and 12 months post-stroke. In the multivariate analysis, significant predictors of OD included advanced age (OR = 1.05, 95%), higher NIH Stroke Scale scores (NIHSS >6; OR = 3.52), and larger lesion volumes (OR = 1.02). In the univariate analysis, additional significant factors included prior strokes (OR = 2.40), poor functional status as assessed by the mRS (mRS >1; OR = 3.13), higher Charlson Comorbidity Index (OR = 1.82), heart disease (OR = 3.15), hypertension (OR = 1.76), and territorial infarction findings (OR = 2.00). Nursing home residence (OR = 4.76) and anterior circulation involvement (TACS, OR = 5.55) were also significant in the univariate analysis. OD was further linked to longer hospital stays (p = 0.049) and an increased likelihood of institutionalization after discharge (OR = 0.47) and sex (OR = 1.52).

Study 10. Arreola V *et al.* [42] followed 247 post-stroke patients (mean age 72.3 ± 11.9 years) at three months following the stroke event in a longitudinal cohort study, assessing OD prevalence and its associated factors with VFS. The prevalence of OD was 39.7% on admission and increased to 41.7% in three months, highlighting the persistence of dysphagia. In the univariate analysis, significant predictors of OD included advanced age (OR = 1.08 per year), absence of previous heart disease (OR = 3.9, 95%), pre-stroke functional dependency as measured by the Rankin score (p = 0.045), lower Barthel Index scores on discharge (p = 0.046), and MN risk (MNA <17, p = 0.033).

Study 11. Espinosa-Val MC *et al.* [7] assessed risk factors for OD in this prospective cohort study on 255 patients with dementia (mean age 83.5 ± 8.0 years) admitted to the psychogeriatric unit at CSDM, with a dysphagia prevalence of 85.9% as determined by the V-VST. Significant risk factors for OD included advanced age, poor nutritional status (MNA <17), low Barthel Index on admission and high dementia severity scales (GDS and FAST).

Study 12. Martín A *et al.* [37] conducted a prospective observational study examining the risk of OD during the first wave of COVID-19 on 205 hospitalized patients (mean age 69.3 ± 17.5 years). Using the V-VST, the prevalence of OD was 51.7%. Significant risk factors identified in multivariate analysis included delirium (OR = 10.97, 95% CI 1.64–73.31, p = 0.013), higher Charlson Comorbidity Index (OR = 1.49, 95% CI 1.02–2.18, p = 0.040), and lower pre-admission functionality as measured by the Barthel Index, with higher functional capacity being protective against OD and lower functional capacity associated with an increased risk (OR = 0.92, 95% CI 0.87–0.98, p = 0.008). In univariate analysis, additional significant associations with OD included older age (p < 0.0001), lower albumin levels (p = 0.025), confusion (p = 0.0001), low mean weight (p < 0.0001), neurological symptoms (p = 0.0001), and lower protein levels (p = 0.025).

Study 13. Viñas et al. [51] evaluated the prevalence of OD and its risk factors in a prospective study on 605 COVID-19 patients across three pandemic waves (mean age 69.2 ± 17.3 years). OD prevalence decreased from 51.7% in wave 1 to 31.3% in wave 2. Significant risk factors for OD included advanced age (OR = 1.04 per year), delirium (OR = 2.38), and MN (OR = 2.71). Severe functional dependency (Barthel Index <40, OR = 3.62) was also a strong predictor. ICU-specific treatments were not significant predictors of OD.

**Figure 1.**
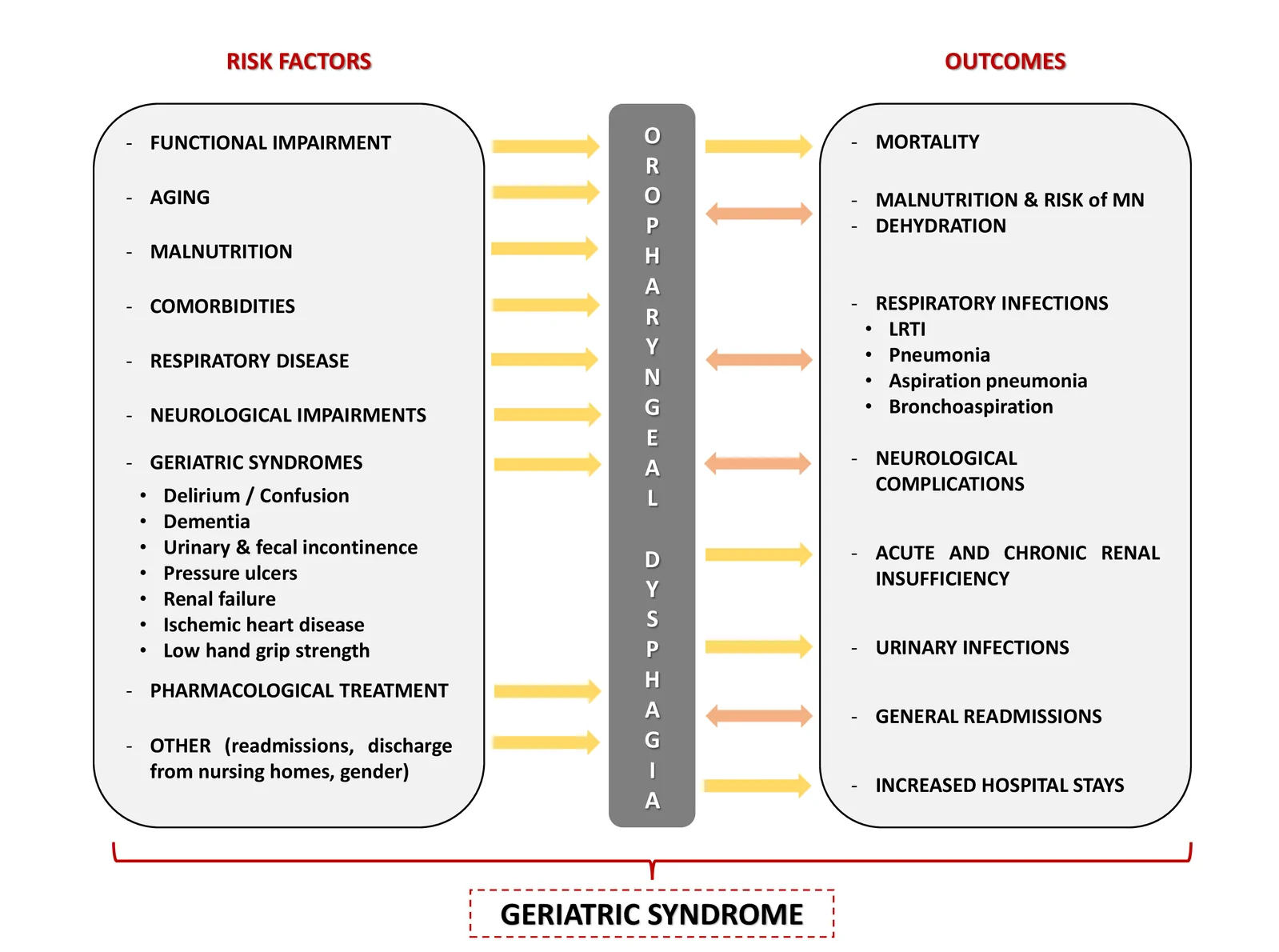
**Risk factors and outcomes of oropharyngeal dysphagia as a geriatric syndrome according to the current findings and those from Karunaratne B *et al* 2024** [9]. *LRTI: lower respiratory tract infections; MN: malnutrition.*

Building on the summaries of the studies, the following section focuses on the 8 main groups of risk factors (reduced functional capacity, advanced age, MN, comorbidities, respiratory disease, neurological impairments, geriatric syndromes and pharmacological treatments) with their variables identified as the main risk factors for OD as a geriatric syndrome (Fig. 1).

Reduced functional capacity was found to be a significant predictor of OD in all thirteen studies across various etiologies with an independent association in 3 of them. In multivariate analysis, impaired functionality (Barthel <100) was found to be an independent risk factor in independently-living older adults, with ORs of 3.35 for OD, 2.76 for impaired safety, and 2.24 for impaired efficacy (3); and in COVID-19 patients (OR= 0.92; p = 0.008; OR= 3.62; p < 0.001) [37,51]. Through univariate analysis, functional capacity (Barthel) was also found to be a predictor of OD in older patients hospitalized with pneumonia and acute illnesses, a Barthel Index <40 was strongly associated with OD in hospitalized patients [43-46], with odds ratios as high as 12.7 [45]. Among frail older patients [48], impaired functional status was linked to significant differences in swallowing safety and efficacy, with lower Barthel Index scores in those with impaired safety (p = 0.002). In independently-living older adults, OD was also strongly associated with low functional capacity, with ORs of 2.24 [3,50]. Similar associations were observed in patients with chronic neurological conditions or acute pneumonia [46], as well as in dementia patients, where a Barthel Index <40 was a significant predictor of OD [7]. In post-stroke patients, severe functional dependency (Barthel Index <40) was identified as a strong predictor of OD, with an OR of 6.12 [42]. Additionally, the modified Rankin Scale (mRS >1) was also found to be a significant risk factor for OD in this population [48].

Advanced age was significantly associated with OD in twelve of the thirteen studies, across various patient populations. Multivariate analysis (3 studies) identified age as an independent risk factor in independently-living older adults (OR= 2.23, p = 0.002) [3,50] and in patients with stroke (OR = 1.05, p <0.0001) [49].

Through univariate analysis, older age was found to be significantly linked with OD in older patients with pneumonia [43,44], in older hospitalized patients admitted in the AGU (OR 1.05, p <0.001) [47] and those with acute illnesses (OR = 1.6, p < 0.001 ) [45], and with chronic neurological conditions or acute pneumonia [46]. Advanced age was also significantly associated with OD in patients with stroke (OR = 1.08 per year, p < 0.001) [42], dementia [7] and in hospitalized COVID-19 patients, both during the first wave [37] and across all three waves (OR = 5.16 per year, p < 0.001) [51].

Malnutrition was found to be significantly associated with OD across seven studies. A low MNA score (MN or at risk of MN) was identified as an independent risk factor for OD in older patients hospitalized with pneumonia (p <0.001) [43,44] and in independently-living older adults (OR: 2.46, p = 0.03) [3]. Univariate analysis also revealed a significant association between MNand OD in COVID-19 patients (OR: 5.16, p < 0.0001) [51]. Similarly, MNwas significantly linked to OD in older patients with chronic neurological conditions [46], dementia [7] and stroke [41].

Additional nutritional markers were also significantly associated with OD. Reduced albumin levels, indicating poorer nutritional status, were observed in patients with dysphagia admitted to the AGU with and without pneumonia (OR 1.90, p < 0.001 ) [44-47], and in COVID-19 patients [7]. In the latter group, low protein level was also significantly associated with OD. Furthermore, lower cholesterol levels and lower BMI were linked to OD in patients with pneumonia [43,44]. A lower BMI, indicative of a lower weight condition, was a risk factor for OD in older patients admitted to AGU (OR 1,47, p < 0.001) [47] and in those with COVID-19 [7].

Comorbidity was evaluated with the Charlson Comorbidity Index across seven studies and was significantly associated with OD through univariate analysis. This association was observed in older patients admitted to the AGU with and without pneumonia (OR 1.16, p < 0.001) [43,44,47], in those with acute and chronic diseases (OR: 1.16, p < 0.001 ; 2017) [45], in patients with stroke (OR 1,22, p<0.001) [49], and in hospitalized COVID-19 patients (OR: 1.49, p = 0.040) [7].

Acute and chronic renal failure were significantly associated with OD in acute hospitalized older patients (OR 1.44, p = 0.003) [45] and in those admitted to the AGU (OR 1.75, p = 0.002) [47]. Similarly chronic kidney disease was linked to OD in hospitalized COVID-19 patients [7].

Using univariate analysis, Ischemic heart disease was associated with OD in patients with pneumonia [44] and stroke (OR 1.78, p = 0.013) [49]. Similarly, the absence of prior heart disease was linked with safe and efficacious swallow in stroke patients (Safety: OR 3.19, p = 0.015; Efficacy OR 4.6; p = 0.019) [42].

Hypertension demonstrated a significant association with OD in univariate analysis. Hypertension was more commonly observed in patients with both stroke and OD (OR 1.74, p < 0.001) [49]. Conversely, it was less frequently reported among patients with OD admitted to the AGU (OR 0.22, p < 0.001) [47].

Neoplasia, considered broadly without referring to a specific type, was significantly associated with OD. According to univariate analysis, a lower prevalence of neoplasia was linked to an increased risk of OD (OR 0.71, p = 0.029) [45].

Respiratory disease was significantly linked to OD through univariate analysis. In older patients admitted to the AGU, a higher prevalence of respiratory disease was linked to a lower risk of OD (OR 0.76, p = 0.049) [47]. Additionally, a high Fine’s pneumonia severity score was associated with OD in older patients admitted with pneumonia [43].

Neurological impairments have been associated with OD through univariate analysis. General neurological symptoms were significantly linked to OD in COVID-19 patients [7]. Neurodegenerative diseases were also significantly associated with OD in older patients admitted to the AGU (OR 2.25, p < 0.001) [47].

Regarding stroke, a history of stroke was significantly associated with OD in patients with chronic disease [46] and in those with stroke [49]. Specifically, among stroke patients, independent factors for OD included stroke severity (NIHSS >6) (OR: 3.52, p = 0.002) and lesion volume (OR 1.02, p= 0.004). Univariate analysis also highlighted significant associations with location, particularly anterior circulation involvement (Oxford Stroke Classification (TACS), OR: 15.52 p < 0.001) and territorial infarction findings (OR 2.20, p = 0.006) [49]. Lastly, univariate analysis highlighted that the presence of major cardioembolic events was associated with OD in stroke patients (OR 2.00, p = 0.014) [49]. Using the same analysis, cerebrovascular diseases were significantly associated with OD in patients admitted with acute illness (OR 1.94, p < 0.001) [45] and in those admitted to the AGU with and without pneumonia (OR 1.88, p < 0.001) [44,45,47].

### Geriatric syndromes

Delirium was a significant risk factor for OD in 3 studies. Martín et al. (2022) reported an OR of 10.97 (p = 0.013) [37], and Viñas et al. (2022) found an OR of 7.09 (p < 0.001) [51], both identifying delirium as a strong independent predictor of OD through multivariate analysis of hospitalized COVID-19 patients. Additionally, univariate analysis revealed an association between OD and delirium in patients with pneumonia [44]. Similarly, the onset or worsening of confusion was associated with OD in patients with both pneumonia and COVID-19 [37,43].

Urinary and fecal incontinence were identified as significant risk factors, with ORs of 4.00 (3.23–4.96) in acute disease settings [45] and in patients admitted with pneumonia [43,44].

Immobility and a history of prior falls were associated with OD, particularly in older adults hospitalized with pneumonia, and acute or chronic illnesses [43-45]. Slower walking rate and weak handgrips were also significantly related to OD [50]. Weak handgrip was also found to be a significant risk factor in patients admitted with chronic disease [46] and in those admitted to the AGU (women OR: 0.84 p<0.001; men OR: 0.89, p < 0.001) [47].

Pressure ulcers were notable contributors to OD risk in hospitalized patients with pneumonia and acute illnesses (OR. 5.92, p<0.001) [43-45].

Dementia was significantly associated with OD in patients admitted to the AGU with pneumonia [44] and without pneumonia (OR 5.93, p < 0.001) [47] and in those with acute disease (OR: 4.69) [45]. Lastly, dementia severity indexes, GDS and Fast, were higher in patients with OD and dementia [7].

Pharmacological treatments were significantly associated with OD through univariate analysis in six out of thirteen studies. Cabré et al. found in two papers, that use of antipsychotics [43,44], antidepressants and benzodiazepines [44] was significantly more common in patients with OD. The use of benzodiazepines was also significantly linked to OD in independently-living older adults [50]. Carrión et al. also reported that the use of sedatives (OR 1.33, p = 0.005), antidepressants (OR 1.46, p < 0.001), and neuroleptics (OR 3.35) increased OD risk in 1,662 older adults hospitalized for acute illnesses [45] and that polypharmacy was more common in patients with chronic diseases [46]. Antidepressants (OR 1.44, p 0.001), drugs against dementia, including cholinergic medication (OR 2.73, p < 0.001) and those for the nervous system (OR 1.62, p = 0.002) were also significantly linked to OD in older patients admitted to AGU [65].

Additional risk factors for OD include: 1) Sex difference that was found to be associated with OD in one study with stroke patients. A univariate analysis suggested that female patients were more likely to develop OD compared to male patients (OR 1.52; p = 0.041) [49]. However, gender and sex were not significant predictors of OD in all the other studies included in this review, [3,7,37,42-48,50,51]. 2) Living in and discharge from nursing homes which were significantly associated with OD in post-stroke [49] and pneumonia-related patients [43,44].

### Secondary findings

#### ROBINS-I-V2 tool and GRADE framework.

The evidence quality evaluation using the ROBINS-I V2 tool (Table 3) identified notable limitations in observational studies assessing risk factors for OD. A primary concern was the reliance on non-gold-standard diagnostics, such as the V-VST (D4: High Risk), which contributed to classification bias. Additionally, inadequate adjustment for confounders (D1: Moderate to Serious Risk) and incomplete use of multivariate statistical analyses (D6: Moderate to Serious Risk) were key weaknesses. While missing data (D5) had a limited impact in cross-sectional and baseline analyses, studies with prospective designs generally exhibited higher methodological rigor compared to cross-sectional studies. Overall, the majority of studies were rated as Moderate (7/13), Low (6/13) or Very Low (1/13) quality.

**Table 3.** Summary of the evidence quality evaluation according to ROBINS-I-V2 criteria.

Study	Study Design	D1	D2	D3	D4	D5	D6	D7	Overall Quality	Key Notes
Cabré et al., 2010 [43] (n=134) °	Prospective Cohort	●	●	●	●	●	●	●	Very Low	Relied on the Water Swallow Test (Domain 4: Critical Risk). Lacked multivariate models (D6).
Rofes et al., 2018 [49] (n=57)	Cross-sectional	●	●	●	●	●	●	●	Low	Used VFS (gold standard) but lacked multivariate analysis (D6). Moderate confounding (D1).
Serra-Prat et al., 2011 [3] (n=254) º	Cross-sectional	●	●	●	●	●	●	●	Moderate	Relied on V-VST (D4: High Risk). Moderate missing data (D5) and limited multivariate analysis.
Serra-Prat et al., 2012 [50] (n=254) *º	Cohort	●	●	●	●	●	●	●	Moderate	Similar to 2011: used V-VST, moderate missing data, limited multivariate analysis.
Cabré et al., 2014 [44] (n=2359) º	Prospective Cohort	●	●	●	●	●	●	●	Moderate	Large sample size. Relied on V-VST and lacked robust multivariate models (D6).
Carrión et al., 2015 [45] (n=1662) º	Multicenter Prospective	●	●	●	●	●	●	●	Low	Incomplete adjustment for confounders (D1) and no robust multivariate models (D6).
Miarons et al., 2016 [47] (n=966) º	Cross-sectional	●	●	●	●	●	●	●	Moderate	Used V-VST, but analysis lacked adjustment for key confounders (D6). Limited robustness in D4.
Carrión et al., 2017 [46] (n=133)	Cross-sectional	●	●	●	●	●	●	●	Moderate	Combined V-VST with VFS, reducing misclassification risk (D4). Multivariate models well-executed.
Rofes et al., 2018 [49] (n=395) º	Prospective Cohort	●	●	●	●	●	●	●	Moderate	Relied on V-VST (D4: High Risk). Moderate adjustment for confounders and statistical analyses.
Arreola et al., 2019 [42] (n=247) º	Longitudinal Cohort	●	●	●	●	●	●	●	Low	V-VST-based diagnosis (D4: High Risk). No robust multivariate models (D6).
Espinosa-Val et al., 2020 [7] (n=255) º	Prospective Cohort	●	●	●	●	●	●	●	Moderate	Diagnosis relied on V-VST, with moderate adjustment for confounders (D1).
Martin-Martinez et al., 2022 [37] (n=205) º	Prospective Observational	●	●	●	●	●	●	●	Low	COVID-19-specific biases the results. No robust multivariate analysis (D6).
Viñas et al., 2022 [51] (n=605) º	Prospective Cohort	●	●	●	●	●	●	●	Low	V-VST-based diagnosis (D4: High Risk). Poor adjustment for confounders and no multivariate models.

º Non-gold -standard diagnostics., *Same sample.

●Critical risk of bias, 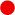 Serious risk of bias, 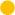 Moderate risk of bias, 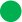 Low risk of bias

**D1:** Risk of bias due to confounding, **D2:** Risk of bias in classification of interventions, **D3:** Risk of bias in selection of participants into the study (or into the analysis), **D4:** Risk of bias due to deviations from intended interventions, **D5:** Risk of bias due to missing data, **D6:** Risk of bias arising from measurement of the outcome, **D7:** Risk of bias in selection of the reported result.

The GRADE evaluation (Table 4) identified strong evidence linking reduced functional capacity, aging, MN, comorbidities, neurological impairments, and geriatric syndromes to increased OD risk, with moderate certainty. Respiratory conditions and pharmacological treatments showed low certainty due to bias and imprecision, while sex differences had very low certainty based on limited data.

### DISCUSSION

This study compiles data from 13 comprehensive and homogeneous studies, involving 7,272 older persons and conducted at CSdM by the same research team and methodology, providing valuable insights into the risk factors associated with OD in older adults with diverse phenotypes. By synthesizing data from different clinical settings, the study identifies 8 major risk factors: reduced functional capacity, advanced age, MN, comorbidities, respiratory disease, neurological impairments, geriatric syndromes and pharmacological treatment as key predictors of OD (Fig. 1). Specific phenotypes including a representative sample of a general population of older patients with OD, such as independently-living older adults, post-stroke patients, those hospitalized for pneumonia, and those with COVID-19 or dementia, also revealed specific risk factors like stroke severity, delirium, functional dependence, MN, and multimorbidity, highlighting the need for phenotype-specific diagnostic and management strategies.

**Table 4.** Summary of the summarized risk factors quality evaluation according to GRADE criteria.

Risk factor	Impact	Nº of participants (studies)	Certainty of the evidence (GRADE)
**Reduced functional capacity**	Strong association between reduced functional capacity (Barthel Index <100 or <40) and increased OD risk across conditions like pneumonia, dementia, and stroke.	~7272 participants [3,7,37,42–51]	**⨁ ⨁ ⨁ ⨀ Moderate** (downgraded for bias)
**Advanced Age**	Advanced age is consistently associated with an increased risk of OD and related complications across various clinical contexts, including pneumonia, COVID-19, and chronic diseases. While some studies lack specific ORs, the observed trends strongly support the biological plausibility of the relationship	~7215 participant. [3,7,37,42-47,49-51]	**⨁ ⨁ ⨁ ⨀ Moderate** (downgraded for bias)
**Malnutrition**	Indicators such as low albumin, BMI, and MNA scores are strongly associated with increased OD risk. Consistent findings across populations and clinical conditions.	~5.158 participants [3,7,37,42-44,46,47,51]	**⨁ ⨁ ⨁ ⨀ Moderate** (downgraded for bias)
**Comorbidity**	Comorbidities such as renal failure, ischemic heart disease, and higher Charlson Comorbidity Index are associated with increased OD risk. Multivariate analysis studies confirm this association, with specific conditions like chronic kidney disease and neurodegenerative diseases showing strong links.	~5763 participants [37,43-45,49,65]	**⨁ ⨁ ⨁ ⨀ Moderate** (downgraded for bias)
**Respiratory disease**	Associations found between pneumonia severity, chronic respiratory disease, LTRI, and bronchoaspiration with increased OD risk.	~1100 participants [43,47]	**⨁ ⨁ ⨀ ⨀ Low** (downgraded for bias, and imprecision)
**Neurological impairments**	Strong associations between neurological conditions (e.g., stroke, neurodegenerative diseases, cerebrovascular disease) and OD risk. Consistent findings across populations.	~5720 participants [7,44,46,47,49]	**⨁ ⨁ ⨁ ⨀ Moderate** (downgraded for bias)
**Geriatric syndromes**	Factors like dementia, delirium, incontinence, immobility, and pressure ulcers significantly increase OD risk. The associations are strong, consistent, and clinically plausible across multiple studies.	~6573 participants [7,37,43-47,50,51]	**⨁ ⨁ ⨁ ⨀ Moderate** (downgraded for bias)
**Pharmacological treatments**	Associations between the use of certain medications (antipsychotics, benzodiazepines, neuroleptics) and increased OD risk. Consistent results across populations.	~5508 participants [43-47,50]	**⨁ ⨁ ⨀ ⨀ Low** (downgraded for bias and imprecision)
**Sex difference**	Women showed an increased risk of OD after stroke in just one study.	~395 participants [49]	**⨁ ⨀ ⨀ ⨀ Very Low** (downgraded for bias, imprecision, Indirectness and inconsistency)

Consistent methodologies for OD diagnosis and data management were employed to confirm its association with aging and functional decline. Central to this study is the V-VST, a validated clinical assessment tool with excellent sensitivity and specificity when performed by trained healthcare professionals (SLP, advanced practice nurses or nutritionist) [53]. While not considered the gold standard like VFS, the V-VST’s accuracy and clinical utility are maximized when performed by skilled professionals. Rigorous data management and statistical methods, including multivariate analyses and odds ratio calculations, also ensure the reliability and credibility of our findings. The methodology underwent peer review, with all studies approved by the Ethics Committee of our institution and conducted following strict clinical research protocols. Additionally, to our knowledge, this is the first study to conduct a large-scale quality of evidence assessment across OD-related research, applying rigorous frameworks such as GRADE and ROBINS-I-V2 to evaluate the strength of our findings. Our results establish a moderate level of evidence within the included studies, demonstrating a commitment to methodological rigor that is often underexplored in OD research [5,66,67]. While this assessment focuses on our dataset, it provides a valuable benchmark for future investigations. Given that most OD studies do not incorporate structured quality assessments, our findings highlight the importance of adopting standardized and transparent methodologies to enhance the reliability of research in this critical area.

To take a closer look at the evidence, our analysis highlights impaired functional capacity as a key predictor of OD, supported by findings from all included studies. Functional decline, assessed using tools like the Barthel Index, Lawton Scale, and Rankin Scale, consistently showed strong associations with OD, regardless of the specific scale employed [14]. Independent associations were observed in 6 of the included studies, with maximal ORs of 12.7 in univariate analyses and 3.35 in multivariate analyses in older patients. This independent risk factor emerged as a critical predictor of OD in our cohort, corroborating findings from prior research [5, 13, 16, 17, 19, 20, 40, 68]. The link between reduced functionality and OD has been consistently demonstrated across studies, highlighting its importance in various clinical settings [14]. Functional impairment is frequently associated with aging, frailty, and other geriatric syndromes [14], although the interplay of contributing factors may vary across individuals and clinical settings. Research by Yang et al (2022) highlights a bidirectional relationship between frailty and OD, with dysphagia significantly increasing the risk of frailty in older adults (OR 3.24; 95% CI 2.51-4.20) [40]. Studies also highlight the high prevalence of OD in frail individuals, emphasizing the need for functional and frailty assessments in geriatric care. Frailty, characterized by reduced physiological reserve and diminished resistance to stressors, resulting in increased vulnerability and adverse outcomes [69], often coexists with dysphagia and sarcopenia, forming a complex interplay. This multifactorial relationship, likely involving age-related muscular degeneration, sarcopenia, SD, and swallowing dysfunction, suggests the need for integrated management approaches in the care of the older adult.

Another relevant key factor was advanced age, with age emerging as a significant predictor in twelve of the thirteen studies [3,7,37,42-47,49-51]. The aging of the world's population has become a relevant issue in today's healthcare perspective, making OD an important geriatric syndrome to consider. As individuals age, the incidence of OD becomes more common, reaching levels of over 80% in acute hospitalized older patients [14,70,71]. Its pathophysiology includes both biomechanical and neurophysiological oropharyngeal sensory and motor neural alterations giving, as a result, an impaired swallow process, putting the patient at risk of severe nutritional and respiratory complications [14,23]. OD in older adults is frequently associated with widespread neurological and structural pathologies linked to the aging process, including cerebrovascular, neurodegenerative, neuro-muscular, or head and neck conditions [6,7,72,73]. As found in our review, the presence of comorbidities was also a relevant risk factor for OD, appearing in 50% of the studies. Additionally, silent age-related cerebrovascular changes, such as leukoaraiosis or small vessel disease, progressively impaired neurological connections on white matter, influencing both the presence and severity of OD [74,75]. Aging itself is also intrinsically linked to OD, with multiple contributing factors implicated in the gradual decline of swallowing mechanisms. These factors include sarcopenia or progressive muscle atrophy [13,76], neuronal loss, and central and peripheral axonal demyelination [77,78] contributing to impaired pharyngeal sensory function, as well as biochemical alterations, such as reduced levels of substance P [79]. This age-related decline supports the concept of a continuous progression from older robust individuals to presbyphagia (preclinical changes) to subclinical impaired biomechanics of swallow response finally leading to impaired safety and efficacy of swallow and ultimately to symptomatic dysphagia in vulnerable older populations such as the ones included in our study [21,80].

MN was also identified as a significant risk factor for OD in nearly 50% of the reviewed studies [3,7,37,42-44,46,47,51]. A study included in this review also showed that OD was an independent risk factor for MN, and both conditions were related to poor outcomes [45]. MN and OD often coexist in a reciprocal cycle: OD impairs swallowing, reducing oral intake and leading to MN, while MN weakens swallowing muscles and exacerbates OD by compromising physiological functions necessary for effective deglutition. Aging further increases the risk of MN due to a reduced basal metabolic rate, loss of muscle mass, and lower physical activity levels [81]. Various studies have shown higher prevalence of OD in the presence of MN, using screening tools like the MNA, its short-form version (MNA-sf), and the NRS-2002, along with clinical and anthropometric evaluations [3,7,37,42-44,46,47,51]. These findings highlight the critical importance of nutritional complications of OD, including dehydration that affects up to 81.2% of hospitalized older patients based on plasma osmolarity values and 78.9% according to the BUN/Cr ratio [82] and is related to chronic renal failure, and the need to perform a nutritional and hydration assessment and intervention in managing and preventing OD in older adults.

Pharmacological treatments were identified as risk factors in six of thirteen studies. Medications with anticholinergic properties, including those for depression, incontinence, epilepsy, and cardiovascular conditions, often impair swallowing through side effects such as dry mouth and extrapyramidal symptoms [15,83]. As found in our patients, neuroleptics were also highlighted in two reviews as potential contributors to dysphagia [47,84].

Several geriatric syndromes were also pointed out as risk factors for OD in this review. Factors such as mobility impairments and immobility (6/13 studies), delirium (3/13 studies), pressure ulcers (4/13 studies), dementia (3/13 studies) and urinary and fecal incontinence (2/13 studies) were significantly associated with OD. Each of these conditions contributes to dysphagia through distinct but interrelated mechanisms. Mobility impairments and immobility lead to muscle atrophy, sarcopenia, and poor postural control, weakening oropharyngeal muscles and increasing aspiration risk [85-88]. Pressure ulcers, as markers of frailty and MN, further impair muscle strength and swallowing efficiency, often exacerbated by prolonged bed rest and reduced nutritional intake [89-91]. Delirium disrupts cognitive and sensorimotor function, delaying swallowing reflexes and increasing aspiration risk [70,89,92,93], while medication such as sedatives and antipsychotics can impair neuromuscular control and/or cause xerostomia [94-96]. Dementia affects voluntary and involuntary swallowing processes, leading to oral residue, aspiration, and MN, worsened by swallowing apraxia, impaired brainstem reflexes, and behavioral disturbances affecting feeding [97-103]. Incontinence signals advanced frailty and neurological impairment, with stroke and Parkinson’s often resulting in concurrent swallowing dysfunction [104-106]. Many older adults with incontinence reduce fluid intake to try to avoid episodes, thereby heightening risk of dehydration and thickened saliva which further compromises bolus transit and swallowing efficiency.

Finally, specific geriatric phenotypes exhibited specific and distinct risk factors for OD. Among stroke patients, OD was significantly associated with stroke severity (NIHSS >6), lesion volume, and anterior circulation infarcts, with female sex also emerging as a potential risk factor [49]. In dementia patients, functional dependency and higher dementia severity scores (GDS, FAST) were notable contributors to OD [7]. For COVID-19 patients, delirium was a strong independent predictor, together with MN and comorbidities such as renal failure [37,51]. Patients hospitalized with pneumonia or acute illnesses showed significant associations between OD and geriatric syndromes like immobility, pressure ulcers, and incontinence [43-45].

OD is a geriatric syndrome with multiple risk factors and associated poor outcomes (Fig. 1) (1). The ESSD-EUGMS position paper [1], of which several researchers involved in the present study were among the authors, set the foundation for further research. The integration of findings from both local and global perspectives underlines the urgent need for early and systematic screening in older adults, particularly those with frailty or multimorbidity. The application of systematic and universal screening tools based on machine learning can generate a binary risk score ("YES/NO") displayed on the clinician's workstation, identifying patients who require further OD evaluation based on their electronic health records. One such tool is the Artificial Intelligence Massive Screening for OD (AIMS-OD) [107]. These tools could significantly enhance early OD detection, reaching larger populations in less time, thereby improving overall management efficiency. Moreover, as recommended by Hu et al, a multidisciplinary approach involving comprehensive geriatric assessments, nutritional interventions, and rehabilitation programs is critical to mitigate the impact of OD on quality of life and healthcare costs [11]. According to recent research, the proper management of older patients with OD could include effective minimal and optimal massive interventions based on fluid adaptation and hydration recommendations (ensure 1500 mL of thickened fluids/day), texture modified diets, nutritional fortification and/or oral nutritional supplements when necessary [108] and improved oral care [82].

### Clinical Implications

These findings have important clinical implications, particularly in geriatric care. The identification of key risk factors such as reduced functional capacity, advanced age, and malnutrition supports the need for systematic and early screening for OD in hospitalized older adults and other at-risk populations. Incorporating AI-massive screening and structured and validated tools such as the V-VST into routine clinical assessments may facilitate timely diagnosis and intervention and reduce the incidence of complications such as aspiration pneumonia, dehydration, and MN. In addition, recognizing the heterogeneity among older individuals and the specific risk profiles of different phenotypes enables the development of tailored care strategies, contributing to more accurate and effective management of OD in real-world clinical settings.

### Study Limitations

Our study has some limitations: while it offers strong and uniform methodology and significant insights, its single-center design may limit the generalizability of findings to other healthcare contexts and patient phenotypes. Additionally, there is minimal variability in diagnostic tools across studies that could introduce bias, although this also enhances the robustness of our synthesis by validating results across diverse methodologies. Another important limitation is the quality obtained from the evaluated studies (most with moderate qualifications) according to the GRADE methodology. This is mainly due to the inclusion of non-randomized clinical trials in the analysis and the use of non-gold standard assessment methods to evaluate and classify patients with and without OD in the included studies. The main reason for this was the nature of the included studies, the majority of which were prospective cohort studies and did not require a randomized design, and the fact that to discriminate patients with and without OD in hospitalized patients, instrumental assessment such as VFS or fiberoptic endoscopic evaluation of swallow (FEES) is not feasible due to the large number of patients evaluated in each study (total cohort ≈7,272 individuals), the potential risk for healthcare professionals (COVID-19 study) and the potential radiation exposure with VFS. In addition, the V-VST was the most commonly used clinical assessment tool (84.61% of studies), has very good psychometric properties for OD (93.17% sensitivity, 81.39% specificity, inter-rater reliability kappa 0.77), and could be administered at the bedside in less than 10 minutes and was performed by expert healthcare professionals [53]. Finally, not all the studies included a multivariate analysis to identify risk factors in a comprehensive manner including ORs and confidence intervals, which limits the statistical precision and interpretability of the findings. Despite these limitations, our results were consistent with those of previous risk factor studies and systematic reviews [5,66,109], which also share several of these constraints. Notably, those studies often address isolated phenotypes or specific risk factors, whereas the strength of our study lies in the unified and accurate assessment of a broad spectrum of risk factors across diverse older phenotypes. Overall, while the single-center design and methodological limitations may influence generalizability and introduce some risk of bias, the consistent diagnostic methods and quality of data management enhance the internal validity of our findings. Additionally, although multivariate analyses were applied in several of the included studies, the potential influence of unmeasured or residual confounding factors cannot be entirely ruled out. Variables such as cognitive status, frailty severity, or complex interactions among multiple comorbidities may have influenced the observed associations. This interplay complicates the ability to establish clear causal relationships and underscores the need for future prospective studies with more comprehensive control of potential confounders.

### Future Perspectives

We are planning future research focused on multicenter collaborations and meta-analytic approaches to quantify pooled effect sizes. To prevent OD and/or its potential complications, primary identification and prevention of key risk factors should be prioritized through public health campaigns and the integration of advanced technologies such as AI-driven screening tools [107]. This identification could further refine risk stratification and intervention planning by facilitating early and large-scale screening and even diagnosis. In addition, studying the long-term outcomes of tailored interventions for OD, particularly in frail populations, would provide critical evidence to optimize geriatric care.

## Conclusion

This study provides a synthesis of OD risk factors in older people, emphasizing their multifactorial nature and clinical implications. Our analysis of a large cohort (7,272 patients) identified eight main risk factor groups, with impaired functionality, aging, and malnutrition as the most critical. Additionally, comorbidities, respiratory diseases, neurological impairments, geriatric syndromes, and pharmacological treatments were also significantly associated with OD.

By leveraging data from single-center research with high accuracy in the diagnosis of OD and data management, it offers unique insights that help advance our understanding of the etiology and management of OD in older adults. Furthermore, this study highlights the importance of distinct phenotypes within the older population, such as those living independently, post-stroke patients, those hospitalized for pneumonia, and patients with dementia or COVID-19, each with unique risk factors that warrant tailored diagnostic and management strategies.

These findings support the consideration of OD as a significant geriatric syndrome and highlight the potential value of targeted prevention strategies and standardized screening protocols based on the early identification of these risk factors. Addressing these factors proactively can significantly improve OD diagnosis, management, and clinical outcomes in older adults. Future research should focus on large-scale, multicenter international studies using systematic review and meta-analytic methods to quantify pooled effect sizes and strengthen the evidence base.
